# Body image dissatisfaction and body regulation practices

**DOI:** 10.1192/j.eurpsy.2022.1490

**Published:** 2022-09-01

**Authors:** E. Nikolaev

**Affiliations:** Ulianov Chuvash State University, Social And Clinical Psychology Department, Cheboksary, Russian Federation

**Keywords:** body regulation practices, Body image dissatisfaction, university students

## Abstract

**Introduction:**

Body image dissatisfaction as negative evaluation of personal physical characteristics is often associated with low self-esteem, eating and affective disorders. What body regulation practices can people resort to when they are dissatisfied with their body image?

**Objectives:**

The goal is to determine body image practices that help people to reduce tension caused by dissatisfaction with their body image.

**Methods:**

We obtained the data by using a focus group technique. The group comprised 43 healthy undergraduate university students of both genders. Afterwards, the data were subject to analysis and systematization.

**Results:**

The findings revealed 11 variants that represent the spectrum of body regulation practices, which semantically can form three groups. The first group combines adaptive regulation practices that help people successfully adapt to actual life situation, develop their capabilities concerning their physiology, personality, intellectual interests, and image making. The second group combines compensatory regulation practices that mainly focus on one of the sides of body regulation, which bear a certain threat to their health (weight control, building up muscle bulk, medical cosmetology procedures, body modification). The third group includes non-adaptive body regulation practices associated with high risk to their health and personal wellbeing (auto-aggressive, hetero-aggressive, and psychopathological).

**Conclusions:**

Information about preferable body regulation practices used by people who are dissatisfied with their body image can help predict health hazards and disorders, as well as work out targeted prevention programs.

**Disclosure:**

No significant relationships.

